# Using Community Feedback to Guide the COVID-19 Response in Sub-Saharan Africa: Red Cross and Red Crescent Approach and Lessons Learned from Ebola

**DOI:** 10.1089/hs.2020.0195

**Published:** 2021-02-18

**Authors:** Eva Erlach, Bronwyn Nichol, Sharon Reader, Ombretta Baggio

**Affiliations:** Eva Erlach, Mag.a iur, is Community Engagement and Accountability (CEA) Delegate and Bronwyn Nichol, MPH, is Community Epidemic and Pandemic Preparedness Delegate, Health and Care Unit; both at the International Federation of Red Cross and Red Crescent Societies (IFRC), Africa Regional Office, Nairobi, Kenya. Sharon Reader, MA, is a Norwegian Capacity (Norwegian Refugee Council) CEA Global Advisor and freelance consultant (previously IFRC Africa CEA Senior Advisor), Glasgow, Scotland. Ombretta Baggio, MA, is Senior Advisor, Community Engagement and Accountability, IFRC, Geneva, Switzerland.

**Keywords:** COVID-19, Risk Communication, Community Engagement, Social Mobilization, Community feedback, Ebola, Infodemic

## Abstract

Risk communication and community engagement are critical elements of epidemic response. Despite progress made in this area, few examples of regional feedback mechanisms in Africa provide information on community concerns and perceptions in real time. To enable humanitarian responders to move beyond disseminating messages, work in partnership with communities, listen to their ideas, identify community-led solutions, and support implementation of solutions systems need to be in place for documenting, analyzing, and acting on community feedback. This article describes how the International Federation of Red Cross and Red Crescent Societies and its national societies in sub-Saharan Africa have worked to establish and strengthen systems to ensure local intelligence and community insights inform operational decision making. As part of the COVID-19 response, a system was set up to collect, compile, and analyze unstructured community feedback from across the region. We describe how this system was set up based on a system piloted in the response to Ebola in the Democratic Republic of the Congo, which tools were adapted and shared across the region, and how the information gathered was used to shape and adapt the response of the Red Cross and Red Crescent Societies and the broader humanitarian response.

## Introduction

Risk communication and community engagement are critical elements of an epidemic response.^1^ Organizations and responders have made great strides to improve the way humanitarian actors listen to the communities they serve. There has been increasing consensus that it is necessary to understand what community members affected by disease are most concerned about, what information they need to protect themselves, and how they perceive response actors.^2^ The actions of community members can end—or sustain—an outbreak, and, therefore, their active support is critical to stop transmission.^3^

Communities' trust in humanitarian responders is key to ending an epidemic,^4,5^ but trust is not easily earned. If communities do not trust humanitarian responders, they will not listen to or act on lifesaving advice and may actively resist efforts to end the outbreak.^6-8^ Communities that do not understand or accept health interventions, or perceive them as a threat, have turned to violence as a means of voicing their concerns, as demonstrated during the Ebola outbreaks in West Africa and the Democratic Republic of the Congo (DRC).^9,10^

To gain communities' trust, work in partnership with communities, and move beyond messaging, it is crucial to continuously understand how communities perceive the disease and the people responding to it. Epidemics evolve quickly, and, therefore, continual analysis and localized, agile response strategies informed by evidence and experience are required.^11^

Despite progress, few regional feedback mechanisms provide information on community concerns and perceptions in real time. The International Federation of Red Cross and Red Crescent Societies (IFRC), together with its member national societies across sub-Saharan Africa, have been working to establish and strengthen such systems to ensure that local intelligence and community insights inform operational decision making. As part of the coronavirus disease 2019 (COVID-19) response, a simple system was set up to collect, compile, and analyze unstructured community feedback from across the region. This system, based on a pilot system in response to Ebola in the DRC, provides relevant and up-to-date information that has been used to adapt national, subregional (eg, West Africa), and regional strategies for responding to the pandemic, making the strategies relevant to the communities they intend to support. In this article, we describe how the system was set up, how tools were adapted and shared, and how the information gathered has been used to shape and adapt the Red Cross and Red Crescent response to COVID-19.

The process of adapting public health interventions requires involvement from all sectors responding to an epidemic, not only those working in the areas of risk communication and community engagement. Platforms are needed to discuss findings and concrete actions across all sectors and partners.^4^ This article also describes how regional interagency community feedback working groups were set up under the lead of IFRC, in coordination with the United Nations Children's Fund (UNICEF).

## Background

Infectious disease emergencies have a disastrous impact at both the community and national levels. Major outbreaks in recent years, including influenza, Zika, Middle East respiratory syndrome, plague, measles, cholera, Ebola, and, most recently, COVID-19, have claimed thousands of lives and highlighted the challenges of engaging communities in epidemic preparedness and response. IFRC and its national societies, active in 192 countries, is well placed to engage in early risk communication and community engagement activities in response to epidemics.

The COVID-19 outbreak reached Africa later than other parts of the world and to date, with relatively milder health outcomes as the number of cases in most countries has remained below the initial predictions.^12,13^ The World Health Organization Regional Office for Africa predicted a prolonged outbreak across the continent, with an estimated 29 to 44 million people infected in the first year, 3.6 to 5.5 million people needing hospitalization, and 83,000 to 190,000 deaths related to COVID-19.^14^ This would overstretch health systems in most countries that already face heavy disease burdens and other disease outbreaks. As a result, the IFRC Africa regional office focused early interventions on risk communication and community engagement, community health activities, and prehospital and clinical care activities. The early focus on risk communication and community engagement was to ensure that national societies in Africa were able to work with communities on preventive measures (eg, handwashing, physical distancing, mask wearing), as community ownership is considered critical to diseases response operations.^1-4^

From the outset of the COVID-19 outbreak, there was an urgent need for clear information about the disease, implications for communities, and response interventions to control the outbreak. Rumors and misinformation about the disease, its origin, and characteristics spread rapidly and led to high levels of suspicion in regard to response interventions.^15,16^ A basic level of understanding about the perceptions of community members was needed to inform national and regional response strategies. Within sub-Saharan Africa, the Red Cross and Red Crescent COVID-19 response focused early efforts on risk communication and community engagement and adapted methods developed by IFRC's community engagement and accountability team to incorporate lessons learned from a large-scale community feedback mechanism designed for the Ebola outbreak in the DRC.

As part of the response to the 10th Ebola outbreak in the DRC, IFRC and the DRC Red Cross, with technical support from the US Centers for Disease Control and Prevention (CDC), established a system to collect, analyze, and act on community feedback relating to the response. More than 1 million qualitative comments from community members were recorded by Red Cross volunteers and added to a central database. The feedback data were coded, analyzed locally, and reported on a regular basis to humanitarian responders working on the different pillars of the Ebola operation. The intention was to make this information available to all responders to encourage its use for operational decision making—a priority that the DRC Red Cross continued to pursue until the outbreak ended.^17^

This was the first time during an epidemic that community feedback was collected and analyzed systematically on a large scale to inform response in real time. While knowledge, attitudes, practices, and perception surveys are commonly used in epidemic responses, the time between traditional data collection and analysis has resulted in the delayed availability of data and findings. Community feedback is commonly heard, shared, and discussed anecdotally, but the IFRC system enabled qualitative community feedback to be quantified.

## Adaptation of a National Ebola Feedback Mechanism

### Establishing a Regional Feedback System

IFRC reviewed and adapted the tools tested and used during the Ebola outbreak in the DRC for the COVID-19 pandemic. The tools included a simple form for recording comments shared during risk communication and community engagement activities such as household visits, focus group discussions, or health promotion campaigns in public places; a guide for conducting focus group discussions; and a simple Microsoft Excel log sheet for entering and managing community feedback. With support from a behavioral science team at the US CDC, IFRC adapted the coding frame developed for the community feedback mechanism for the Ebola operation in the DRC for COVID-19 feedback data.

### Sources of Country-Level Feedback

Red Cross and Red Crescent national societies in Africa have different programs and different levels of experience and capacity. Accordingly, it was essential to identify which community feedback channels were already in use, which activities allowed for an integration of a community feedback component, and which new ways of collecting community feedback could quickly be introduced. Some national societies already had a functioning feedback mechanism in place, such as the system to record community feedback during risk communication and community engagement activities in the DRC, whereas other national societies did not have such experience. Nevertheless, of the 48 national societies that had the capacity to respond to the COVID-19 pandemic in sub-Saharan Africa, as of December 2020, 40 have collected and shared community feedback.

New ways of gathering almost real-time community feedback, including rumors, observations, beliefs, questions, suggestions, sensitive and violent comments, as well as praise and acknowledgment, have been discussed and introduced, and IFRC provided guidance to national societies to ensure specific complaints are treated in an appropriate and timely manner. Many national societies began collecting community feedback during health promotion activities. For those contexts where direct, in-person contact was not possible due to lockdowns imposed by governments, WhatsApp groups with community members or community volunteers were established to receive feedback and address rumors, questions, suggestions, or concerns. National societies also scaled up their use of social media, such as Facebook, to engage with community members about COVID-19. No personal information apart from demographic variables are recorded and respondents cannot be identified.

### Coding and Analyzing Feedback Data

Many national societies have been sharing their feedback data with the IFRC Africa regional office, where the data are cleaned, coded, and analyzed. The sharing of these data was harmonized by providing simple Excel log sheets for managing the qualitative feedback. Most of the data are recorded in local languages, translated and entered by country-level staff into Excel log sheets in English or French, and shared with the Africa regional office by email. In countries that do not have systems to collect primary feedback data or where data are available only in languages other than English or French, primary feedback data are combined with trends reported by focal points. Between January and October 2020, more than 110,000 individual community feedback comments from 25 countries were shared and analyzed together with highlights from 15 additional countries. The most feedback data were shared by the DRC Red Cross (83%), followed by Cameroon Red Cross (10%) and Sierra Leone Red Cross (2%).

**Figure 1.**
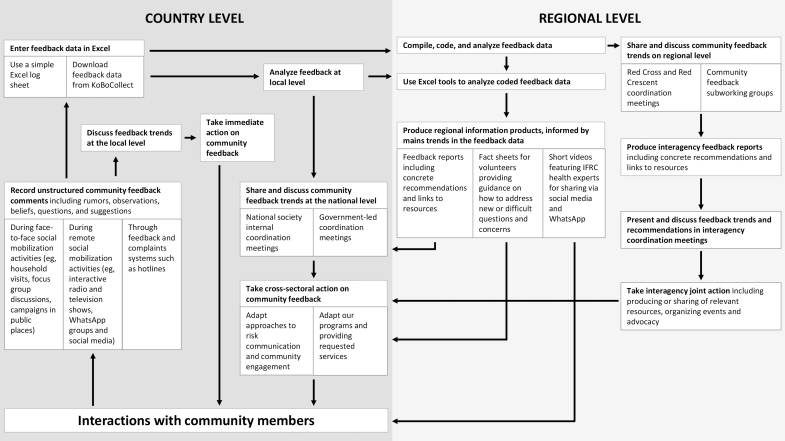
Flow chart of information and actions needed to use community feedback to inform the COVID-19 response at national and regional levels in sub-Saharan Africa. Abbreviation: IFRC, International Federation of Red Cross and Red Crescent Societies.

For the regional analysis, a weighting strategy was used to account for differences in the amount of data shared by countries and the different feedback channels used for data collection. An Excel dashboard was developed to facilitate the process of interpreting and reporting on the coded feedback data. This dashboard can be accessed by the regional COVID-19 operations team, and dashboards containing country-level data are shared with the country teams. Figure 1 presents a flow chart of the information and actions needed to use community feedback to inform an epidemic response at national and regional levels.

**Figure 2.**
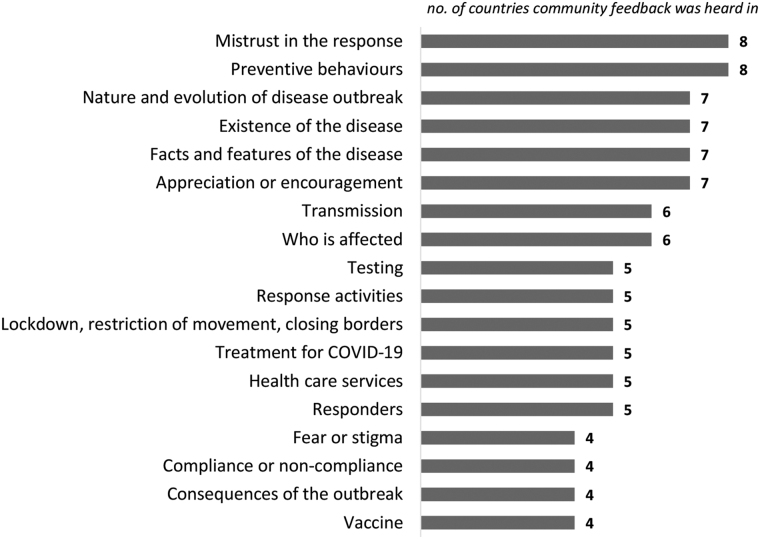
Most common topics heard across 9 sub-Saharan African countries that shared community feedback during COVID-19 response, September 21 to October 4, 2020. This chart includes topics heard in 4 or more countries.

On a regular basis (initially every week and then every 2 weeks), the IFRC Africa regional office produces simple reports on trends in community feedback, with recommendations on how to address the main concerns. Figure 2 shows a graph of the most common feedback topics across countries; graphs like this are included in biweekly regional community feedback reports. This regular analysis of feedback informs the development of information products to address rumors and concerns and respond to communities' most frequent questions. A fact sheet produced by IFRC (or the IFRC Africa regional office) provides simple and clear answers for staff and volunteers is produced and shared along with the feedback report. Short videos featuring health experts are produced and shared via social media and WhatsApp, with themes selected from the most common feedback topics. The fact sheet and videos, available in English and French, provide staff and volunteers with strategies for answering difficult questions and give clear advice on how to stay safe.

## From Feedback to Action

### Adapting Approaches to Risk Communication and Community Engagement

One of the first trends observed in the community feedback data was that many people believed COVID-19 was not a big threat and that governments were using the pandemic to push their own political agendas. For example, one person stated, “COVID-19 is not a big deal, but the ruling party or the government used it to divert the political view and opinion of the people toward the election after the coming 2 months” (community feedback shared with Ethiopia Red Cross, May 2020).

As information provided by the government was not trusted, national societies had to find ways to identify those who were trusted by communities and work with them to share needed information. For example, the retired Ethiopian marathon runner, Haile Gebrselassie, spoke openly about the COVID-19 crisis, shared information about the pandemic, and called for joint action to halt the disease. It became increasingly clear that many community members did not trust the government to provide correct information about the epidemic. One person claimed: “There are many COVID-19 positive cases that the government doesn't publish” (community feedback shared with Burundi Red Cross, June 2020).

To ensure community members receive accurate information about COVID-19, Burundi Red Cross has been working with community leaders to mobilize people in their communities. During meetings with community leaders, Red Cross volunteers share updates on the epidemic and answer questions. Community leaders, who are often more trusted in their communities than the government, then share the information and advice about protective behaviors with their communities.

Another clear theme that emerged across the countries was that COVID-19 does not pose risks to those who are not white or rich. These comments are often combined with mentions of never having seen anyone sick with the disease, for example: “COVID-19 does not kill black people” (community feedback shared with Zimbabwe Red Cross, July 2020).

To ensure that community members are aware of the risks associated with COVID-19, national societies adapted their approach to risk communication and community engagement by organizing interactive TV and radio shows, during which community members call in, share concerns, and ask questions. Some national societies, such as Ghana Red Cross, worked with the community to produce testimonials from recovered people to address misbeliefs that those who had the disease could still infect people, or that they are telling lies to receive money.

### Using Community Insights to Inform an Effective Response

Knowledge of key prevention methods among community members increased over the course of the outbreak,^18,19^ but lower-income households questioned the practicality of these approaches due to insufficient resources. The following type of feedback was common: “Vulnerable households cannot respect handwashing as it is difficult for them to find water and buying soap or gel is not their priority” (unpublished national society report, Madagascar, May 2020).

To address this challenge, national societies supported communities to find affordable, practical solutions. In Botswana and the DRC, teams of volunteers supported communities to construct traditional handwashing stations, using poles and plastic containers provided by the community. These were placed at entrances to busy places to make it easy for people to wash their hands. South Sudan Red Cross also conducted water, sanitation, and hygiene activities as a response to needs voiced by communities and provided handwashing items (eg, soap and buckets) to community members. The appreciation for these activities was recorded in the feedback data, such as this: “Thanks to SSRC [South Sudan Red Cross] for repairing boreholes for us” (focus group discussion, South Sudan, July 24, 2020).

During their activities, Red Cross and Red Crescent staff and volunteers noted that community members suffered from anxiety and stress, especially related to finances, caused by restrictions imposed to reduce the spread of COVID-19. Feedback shared with Cameroon Red Cross on September 19, 2020 included, “How to cope with stress during this pandemic?”

To ensure that volunteers can effectively cope with people's precarious mental states, national societies trained volunteers on how to provide psychological first aid. National societies in Kenya and Cameroon support national call center services to provide psychosocial first aid to those who need it.

### Advocating for Communities' Needs

While some community feedback can be addressed directly by national societies, the solution often lies outside of the Red Cross and Red Crescent mandate. When sharing community feedback trends in national and regional coordination structures, it is therefore critical that the Red Cross and Red Crescent shares the suggestions and needs voiced by communities and advocates for addressing them. For example, Cameroon Red Cross staff and volunteers repeatedly heard requests for water in order to practice proper hygiene, such as this feedback shared from September 2, 2020: “For us to be able to wash our hands, the government should install a borehole in the village.” These suggestions were shared and discussed with the Cameroon national coordination structure for the COVID-19 response. As a result, access to water in vulnerable communities was improved through the construction of 2 boreholes (to extract water from the ground) and the installation of water tanks in at-risk communities with a regular supply of water by the national water company.

Another common feedback topic was criticism of politicians, military, and police officers not wearing face masks or practicing physical distancing. One participant from a focus group discussion held August 25, 2020 in Cote d'Ivoire stated, “The government doesn't respect prevention measures, so we don't respect prevention measures either.”

This feedback was shared and discussed in coordination meetings at national and subregional levels. Volunteers received updated talking points and it was discussed how to best address communities' concerns and doubts. The South Sudan Red Cross shared these concerns with the police, discussed their responsibility as role models in the fight against the pandemic, and provided face masks to them.

### Interagency Coordination

One of the key lessons learned during the response to the Ebola outbreak in the DRC was that collecting community feedback and using it only internally to adapt messages was not enough. To drive action, this information also needs to be shared and discussed among all the different sectors of the response. Taking action is not limited to using information to inform health promotion campaigns, it also includes adjusting programs such as providing requested services or changing how the program works.

To provide a platform for discussion across all sectors and partners involved in the response to COVID-19, interagency community feedback working groups were set up under the lead of IFRC and UNICEF. These subgroups were created under the broader technical working groups for risk communication and community engagement: 1 in the East and Southern Africa and 1 in the West and Central Africa UNICEF regions, which guided the interagency coordination structure. The concept of these groups had been piloted during the Ebola response, with the aim to analyze, share, and encourage action on the most frequent trends in community feedback across agencies. The trends shared by partners inform specific recommendations addressed to the relevant pillars of the response at both national and regional levels. Following these recommendations, partnerships were established between the risk communication and community engagement working group and other technical working groups, guidance notes and fact sheets were developed, and media dialogues with local journalists were organized.

## Discussion

Responses to epidemics, including COVID-19, illustrate the need to find ways to build mutual trust, effectively engage in meaningful dialogue, collaborate with communities and local leaders, and adjust interventions over time based on the feedback and perceptions of affected and at-risk communities.

To react quickly and early to COVID-19 in sub-Saharan Africa, a basic approach was chosen in which a minimum amount of information is collected across the countries. The system needs to be strengthened further and the different components of the mechanism at both the national and regional levels need to be refined and improved over time in order to increase the amount of feedback where numbers are low and to respond to community feedback in a more systematic and effective way.

Although regional analysis is important, most concrete action should (and often only can) occur at national or even district levels. While the current feedback mechanism has been coding and analyzing community feedback at the regional level, qualitative information can be better analyzed and interpreted at the local level where contextual knowledge is readily available. Ongoing efforts exist to enhance the analytical capacity of those managing community feedback within national societies at the country level, which can lead to a faster local response to community feedback. A series of webinars has been conducted and country-level colleagues have started coding and analyzing their own feedback data, using the same harmonized tools used at the regional level.

One of the biggest challenges in the current approach to recording community feedback is the inability to link different feedback comments to demographic variables such as gender, age, or other factors. Paper forms are often used as a practical way to record feedback in areas where smartphones are not available. To save paper, 1 form is used for multiple activities, and feedback comments are recorded without linking each comment to specific demographic variables. As feedback is often collected during activities such as household visits or discussions with more than 1 person, the feedback comments are again not linked to a specific gender or age, as these variables might be mixed in the group of people providing the information. Consequently, an analysis of differences in perceptions between demographic groups is often not possible, preventing an understanding of the nuances of the situation.

Data collection is part of regular risk communication and community engagement activities, not a standalone activity, and, therefore, a sampling approach is not applied. Feedback is often collected during household visits, interactive radio shows, social media, or WhatsApp. Because it is not a specific proportion of community subgroups providing the feedback, findings are not statistically representative of the communities where they were collected. Due to the open method of recording comments during risk communication and community engagement activities, on social media, or during interactive radio shows, and the lack of a structured sampling approach, analyzing trends over time across a country or region is difficult, as the area and population of those sharing feedback changes over time.

By not collecting demographic details and names, the process is kept light and easy to manage for volunteers. By not sampling, national societies were open to hearing feedback through a broader range of channels. The current approach is thus a balancing act between ensuring all the information received by community volunteers is documented and having detailed demographic data and robust sampling. Triangulation with social science data and proactive ways of systematically monitoring community perceptions through structured surveys can further strengthen the system.

Although advances have been made to improve the coordination of community feedback and sharing trends with decision makers, responding to community feedback is still often seen as the sole responsibility of those working on risk communication and community engagement. This work is often siloed and used only to adapt risk communication and community engagement approaches or to adapt messages provided to communities. More ownership among leaders at country and regional levels can ensure a more holistic and cross-sectoral response to community feedback.

## Conclusion

In a December 2019 resolution of the Council of Delegates, the Red Cross and Red Crescent national societies committed to “systematically listening to, responding to and acting on feedback from the people and communities we aim to serve.”^20^ The IFRC community engagement and accountability strategy for Africa recommends making it mandatory that a feedback and complaints system is established and functioning within all programs funded by partner national societies and IFRC.^21^

It is, therefore, a priority for Red Cross and Red Crescent national societies to ensure that all programs and operations include a basic feedback component. As discussed, the COVID-19 operation has provided an opportunity and underscored the urgency to take a big leap toward these objectives. The COVID-19 response showcased how simple ways to systematically listen to communities and respond to them can be integrated into an operation and harmonized across sub-Saharan Africa. It has also shown that specific actions can be taken to respond to community feedback at local and regional levels, and interagency coordination of community feedback can drive action. This regional system will provide a good basis for further integrating feedback mechanisms in programs and operations beyond health emergencies.

Bringing up-to-date community perspectives to decision makers takes time and requires a coordinated effort, financial sustainability, and long-term support to strengthen the capacity of local organizations to roll out a high-quality system at scale, including in future health crises. Sustained investment in feedback systems for emergency responses will ensure a feasible and accepted practice of systematically using sociobehavioral data to shape strategies and interventions according to emerging evidence over time.
